# Evaluation of Total Serum IgE Level and Associated Factors in Asthmatic Patients at the University of Gondar Hospital, Northwest Ethiopia: A Comparative Cross‐Sectional Study

**DOI:** 10.1002/hsr2.71711

**Published:** 2025-12-29

**Authors:** Ayenew Assefa, Tadelo Wondmagegn, Markos Negash

**Affiliations:** ^1^ Department of Medical Laboratory Science, College of Health Science Debre Tabor University Debre Tabor Ethiopia; ^2^ Department of Immunology and Molecular Biology, School of Biomedical and Laboratory Sciences University of Gondar Gondar Ethiopia

**Keywords:** asthma, asthma severity, cholesterol level, helminth infection, total serum IgE

## Abstract

**Background and Aims:**

Asthma is a chronic respiratory disorder with both genetic and environmental underlying risk factors. Allergic asthma, the most prevalent kind, is mostly ascribed to aberrant T helper type 2 (Th2) inflammation and is brought on by allergens. Immunoglobulin E (IgE) is known to play a critical role in asthma and also has a main function in immunity to parasites such as helminths. In this study, we aimed to assess the total serum IgE level and associated factors among asthmatic patients at the University of Gondar Hospital, Gondar, Northwest Ethiopia.

**Methods:**

A comparative cross‐sectional study was conducted from June to August 2019 at the University of Gondar Hospital among asthmatic patients. A structured questionnaire was used to collect the sociodemographic characteristics of patients. Venous blood and stool samples were collected from participants to determine outcomes and clinical factors. The data was analyzed using SPSS version 20. ANOVA, the Kruskal–Wallis test, the Mann–Whitney test, Spearman's correlation, and binary logistic regressions were used in data analysis. A *p* value ≤ 0.05 was considered statistically significant.

**Results:**

A total of 88 study participants were included. The geometric mean of total IgE level was 366.6 ± 5.4 and 52.2 ± 3.6 IU/mL for patients and controls, respectively (*p* < 0.001). The geometric mean total IgE levels of patients with positive and negative helminth infections were 1282.9 ± 3.6 and 294.4 ± 5.6 IU/mL, respectively (*p* = 0.237). There was no significant difference between the dependent variable and the independent variables.

**Conclusion:**

This study showed significantly higher total IgE levels in asthmatics than in controls. The assessment of total IgE level in asthmatics, particularly in those with allergic asthma, is a direct measurement of airway inflammation, and thus, the estimation of total IgE level should be included in routine practice.

**Abbreviations**
ABEI: amino butyl ethylisoluminol
BHR: broncho hyperresponseveness
BMI: body mass index
BT: biotechnica
CI: confidence interval
EOS: eosinophils
FITC: fluorescein isothiocyanate
GINA: Global Initiative for Asthma
HDL‐C: high‐density lipoprotein‐cholesterol
IgE: immunoglobulin E
IL: interleukin
IU: international unit
LDL‐C: low‐density lipoprotein‐cholesterol
OR: odds ratio
SWG: syringe width gauge
TC: total cholesterol
TG: triglyceride

## 1 Background

**Figure 1:**
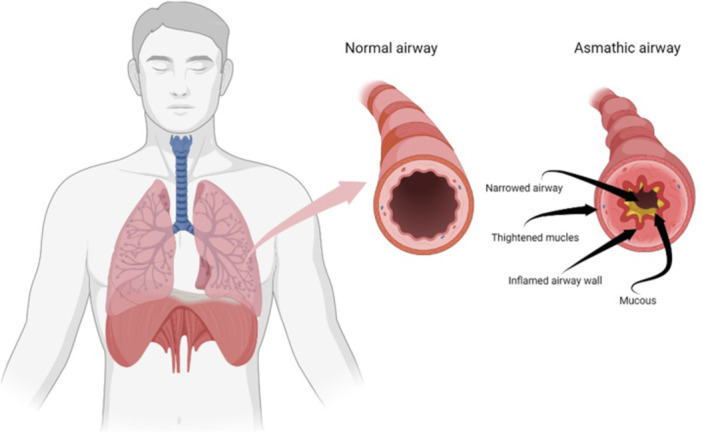
Asthmatic airway alterations. Basis of preventive and non‐pharmacological interventions in asthma [4].

Asthma is a common chronic illness of the airways [1], characterized by three major features: (i) reversible and intermittent airway blockage resulting in recurrent bouts of symptoms like coughing, dyspnea, chest tightness, and wheezing; (ii) Broncho hyperresponsiveness (BHR), which is defined as hypersensitivity to bronchoconstrictors like histamine and cholinergic agonists; and (iii) inflammation of the airways [2]. BHR, which is a vital clinical feature of asthma, results from a complex and multifaceted interaction between the hypercontractility of airway smooth muscle, inflammation within the airways, and the remodeling of the airway structure (Figure 1). More vividly, BHR is caused by a heightened sensitivity to direct pharmacologic bronchoconstrictor stimuli, such as inhaled histamine and methacholine, or to endogenous mediators that are released by activated airway cells, particularly mast cells [3].

Asthma is typically divided into two categories: intrinsic and extrinsic asthma. Extrinsic asthma, the most prevalent kind and commonly referred to as allergic asthma, is ascribed to aberrant T helper type 2 (Th2) inflammation and is brought on by allergens. Many things, on the other hand, can cause intrinsic asthma, including aspirin, lung infections, exercise, cold, stress, obesity, and more [5]. Allergic asthma, also considered to be the classic TH2 lung disease [6], starts with the processing of inhaled antigens by dendritic cells, a subset of antigen‐presenting cells (APCs) in lung tissue, and their presentation to T lymphocytes (results in activation of T cells) via the interaction of their ligand, CD80 (B7‐1), and the receptor molecule CD28 on T cells. In addition, cytokines originating from epithelial cells encourage Th2 cell activation and polarization [7, 8].

In response, TH2 lymphocytes release a range of proinflammatory cytokines. These cytokines include IL‐4, which mediates class switching of B cells and synthesis of immunoglobulin E (IgE); IL‐5, which supports airway eosinophilia; and IL‐13, which mediates hyperresponsiveness of the airway through its impact on airway smooth muscle cells. Besides, IL‐4 and IL‐13 have a direct action on the airway epithelium, causing goblet cell metaplasia and an overproduction of mucus. On the other hand, IgE antibodies possess receptors on mast cells, and when an allergen cross‐links with specific IgE attached to these cells, it initiates degranulation. This degranulation of mast cells leads to the release of mediators such as histamine, which cause symptoms including vasodilation, contraction of bronchial muscles, and heightened mucus secretion. These cytokines, and cytokines such as IL‐3 and IL‐9, also activate endothelial cell adhesion proteins, intracellular adhesion molecule‐1 (ICAM‐1), and vascular cell adhesion molecule‐1 (VCAM‐1), which help move inflammatory cells from blood vessels into the airway. All of the above‐listed events initiate the allergic cascade, resulting in symptoms and clinical conditions of asthma, particularly allergic asthma [8, 9, 10, 11]. The severity varies greatly amongst patients; some people have a very serious condition, while others only have minor symptoms [12].

The function of IgE antibodies in allergy and asthma is well‐established. Elevations in IgE antibody levels in the bloodstream are frequently linked to asthma (commonly allergic asthma), and family genetic analyses have demonstrated a relationship between BHR and IgE levels. The primary mediator between IgE and BHR has been suggested to be mast cells. FcεRI‐mediated cross‐linking of IgE bound to mast cells initiates the degranulation and release of preformed vasoactive mediators [13] such as histamine, prostaglandin D2, and leukotriene C4. These mediators can induce mucus secretion, mucosal edema, and bronchoconstriction [14]. IgE antibodies can also bind to FcεRI receptors on basophils, dendritic cells, endothelial cells, eosinophils, and airway smooth muscle cells [15]. Overall, IgE antibodies, both specific and total, are significant predictors of asthma [16].

Increased IgE levels are also observed in inflammatory diseases, primary immunodeficiency diseases, and parasitic infections (such as strongyloidiasis, ascariasis, and schistosomiasis) [17, 18]. Helminth infections have an active immunomodulation effect and shift towards TH2 responses [19]. Geohelminths, or soil‐transmitted helminths, induce aberrant natural immune development, leading to a shift in the immune response to the Th2 type that affects the development of asthma. Smoking and cholesterolemia can also influence the onset and risk of asthma. Raised blood cholesterol levels are becoming an increasingly serious health issue in many developed and developing countries. Over the years, a number of reports have indicated that cholesterol plays a significant, possibly even unique, role in pulmonary physiology. Smoking is another factor that aggravates asthma and increases the morbidity, severity, and reduced lung function associated with asthma [20]. Therefore, this study aims to assess the total serum IgE levels in asthmatic patients attending the outpatient clinic of the University of Gondar Hospital, Gondar, Ethiopia, with the purpose of comparing total IgE among controls and patients as well as identifying associated factors.

## 2 Methods

### 2.1 Study Area

This study was carried out at the University of Gondar Hospital, Amhara region, north‐western Ethiopia. According to a 2007 Ethiopian Central Statistical Agency (CSA) office report, Gondar is located 747 km away from Addis Ababa and has 206,987 inhabitants [21]. The hospital is a 400‐bed university hospital that provides care to four million people in the surrounding area. Approximately 3500 chronic patients are served annually by the hospital's chronic unit clinic. The clinic includes patients from medical, emergency, surgical, and other wards as well as those with diabetes mellitus, heart failure, rheumatoid arthritis, hypertension, chronic liver disease, renal disease, and asthma.

### 2.2 Study Design and Period

Patients diagnosed with asthma by physicians and who had been followed in the chronic unit clinic were recruited. Patients were not identified as having either allergic or non‐allergic asthma. Patients were classified as mildly persistent, moderately persistent, severely persistent, and intermittent based on the duration of their symptoms and the minimum amount of maintenance therapy needed to bring them under control. Patients were also divided into three age groups: 18–40, 40–70, and > 70 [22]. We did not recruit patients with asthma who had other chronic infections. As a control, we used serum from available blood donors who are healthy and have no known clinical condition. The age range of the controls was from 19 to 30 years.

### 2.3 Study Population

The study included all patients with physician‐confirmed asthma who were monitored at the chronic unit clinic during the study period.

### 2.4 Variables

The outcome variable of this study was total serum IgE level, and the independent variables were sociodemographic characteristics and clinical factors such as helminth infection, cholesterol level, severity of asthma, family history, and smoking status.

### 2.5 Sample Size and Sampling Technique

The statistical formula *n* = (*z*_(__1_ − *α*_/2)_)^2^ × *P* (1 − *P*)/*d* ^2^ was used to calculate the sample size. We take into account a 95% confidence interval, a 3% (0.03) margin of error, and the 2.0% asthma prevalence from the World Health Survey study conducted in Ethiopia [23]. A sample size of 88 was taken based on this assumption.

### 2.6 Data Collection

Sociodemographic information, as well as factors like physical activity, smoking, family history, exacerbations, and history of hospital admission, was gathered using a structured questionnaire. Laboratory technicians collected stool and blood samples. The investigator and laboratory technologists conducted laboratory investigations for the clinical factors (helminth infection, serum cholesterol level) and outcome variable (serum IgE level measurement).

### 2.7 Laboratory Methods

In total, 4 mL of blood sample was drawn from the study participants. Serum was collected and left at −20°C until analysis. Total IgE level was determined using a Maglumi 800 IgE analyzer. The specific IgE concentrations to allergens were not determined.

The Maglumi 800 analyzer operates on the basis of chemiluminescence immunoassay. It uses an anti‐IgE monoclonal antibody to label ABEI and another monoclonal antibody to label FITC. The procedure is as follows: an ABEI label, an FITC label, a control, a calibrator, a pre‐diluted sample, and anti‐FITC‐coated magnetic microbeads are mixed and incubated at 37°C to form a sandwich. Then, the supernatant is decanted, and cycle washing is performed once. Subsequently, the starter reagents are added, and a flash chemiluminescent reaction is initiated. The light signal is measured by a photomultiplier as RLU (relative luminescence units) within 3 s and is proportional to the concentration of IgE present in controls or samples [24].

We used a BT 2000 analyzer to measure the cholesterol level. The principle of the BT 2000 analyzer is based on the fact that the cholesterol in the sample begins as a colored complex following chemical addition and a sequence of reactions. The intensity of the color formed is proportional to the cholesterol concentration in the sample. To detect helminth infection, stool samples were subjected to both the direct and the formol‐ether concentration techniques.

### 2.8 Quality Control

Standard reagents and materials, a pre‐tested questionnaire, appropriate sample collection procedures, routine supervision, and follow‐up of the data collectors were carried out. In the analytical phase, standard operating procedures (SOPs) were followed, and quality control was conducted to validate the instruments' (BT 2000 and Maglumi 800 analyzer) performance. In the post‐analytical phase, every transactional measure was examined, and the outcomes were precisely documented.

### 2.9 Data Analysis and Interpretation

IBM SPSS Statistics version 20 was used to enter and analyze the data. Descriptive statistics were used to summarize the characteristics of the study population. For the analysis of normally distributed variables, ANOVA was utilized, and for non‐normally distributed variables, logistic regression and the non‐parametric tests (Kruskal–Wallis test, Mann–Whitney test, and Spearman's correlation) were employed. A *p* value of < 0.05 was considered statistically significant.

### 2.10 Ethical Consideration

The Ethical Review Committee of the School of Biomedical and Laboratory Sciences approved the protocol for the study. A letter of support was written to the University of Gondar Hospital. All participants received information about the study from the data collectors and were assured of the confidentiality and anonymity of their data. Finally, written informed consent was obtained from the participants before data collection.

## 3 Results

### 3.1 Sociodemographic Characteristics

The study included 88 participants in total: 22 controls (54.5% female) and 66 patients (71.2% female). The mean age of patients was 53.4 years, ranging from 20 to 84 years, and that of controls was 22.8 years, ranging from 19 to 30 years. A total of 48 out of 66 (72.7%) were urban residents. The majority of the patients were in the age range of 40–70 years, while all controls were in the age range of 18–40 years (Tables 1 and 2).

**Table 1:** Sociodemographic characteristics of the patients at the University of Gondar Hospital, Northwest Ethiopia, 2019.

Patients	Number	Percent (%)
Age		
18–40	12	18.2
40–70	48	72.7
70+	6	9.1
Gender		
Male	19	28.8
Female	47	71.2
Residence		
Urban	48	72.7
Rural	18	27.3
Total	66	100

**Table 2:** Sociodemographic characteristics of the controls at the University of Gondar Hospital, Northwest Ethiopia, 2019.

Controls	Number	Percent (%)
Age		
18–40	22	100
40–70	—	—
70+	—	—
Gender		
Male	10	45.5
Female	12	54.5
Total	22	100

### 3.2 Total Serum IgE Levels in Controls and Patients

The geometric mean total serum IgE level for the patients was 336.6 ± 5.4 IU/mL, with a median of 456.9 IU/mL. The values ranged from 1.0 to 3200 IU/mL, which varied considerably (coefficient of variation: 86.9%). The 25th percentile was 105.4 IU/mL, and the 75th percentile was 1410 IU/mL. Similarly, the geometric mean total serum IgE level for the controls was 52.2 ± 3.5 IU/mL, with a median of 63.07 IU/mL. The values ranged from 3.09 to 811.1 IU/mL. The 25th percentile was 23.7 IU/mL, and the 75th percentile was 106.6 IU/mL. There were significantly higher levels of total IgE in the patients as compared to the controls (*p* < 0.001).

Female patients had lower IgE levels with a geometric mean of 287.8 ± 4.9 IU/mL versus 495.8 ± 6.7 IU/mL for males (*p* = 0.131), and in controls, males had lower levels with a geometric mean of 41.6 ± 3.8 versus 63 ± 3.4 IU/mL for females (*p* = 0.742). However, median values were higher in males than in females for both patients and controls.

There was no statistical correlation identified between age and levels of IgE in patients (*p* = 0.911) or controls (*p* = 0.918). The geometric mean total IgE levels were 177.3 ± 9.3, 434.6 ± 4.4, and 156.9 ± 6.3 IU/mL in the 18–40, 40–70, and 70+ age groups of patients, respectively (*p* = 0.263). The median total IgE level peaked at 509.3 IU/mL in the 40‐ to 70‐year age group and 200.2 IU/mL in the 18–40 age group and decreased, reaching 127.3 IU/mL in the group above 70 years. The geometric mean total IgE level was 337.7 ± 6.9 and 336.2 ± 5.1 IU/mL for rural and urban resident patients, respectively (*p* = 0.852) (Table 3).

**Table 3:** Total IgE levels in the study participants with respect to sociodemographic characteristics and associated factors.

		Total IgE (IU/mL)			*p*
Patient characteristics	*N*	Min	G. mean	Max	
Overall	66	1.0	336.6 ± 5.4	3200	
Age (years)					0.263
18–40	12	1.0	177.3 ± 9.3	2079.8	
40–70	48	13.34	434.6 ± 4.4	3200	
70+	6	30.67	156.9 ± 6.3	2907	
Gender					0.131
Male	19	13.34	495.8 ± 6.7	3200	
Female	47	1.0	287.8 ± 4.9	3200	
Residence					0.852
Urban	48	1.0	336.2 ± 5.1	3200	
Rural	18	13.34	337.7 ± 6.9	3200	
Severity					0.324
Intermittent	8	1.0	399.7 ± 14.9	2590.6	
Persistent mild	35	21.46	305.6 ± 4.2	3200	
Persistent moderate	11	15.99	567.1 ± 5.9	3200	
Persistent severe	12	13.34	246.8 ± 5.2	2595.7	
Smoking					0.936
Nonsmoker	62	1.0	338.7 ± 5.5	3200	
Ex‐smoker	4	40.31	305.4 ± 6.6	3200	
Family history					0.74
Yes	11	31.35	164.4 ± 3.3	978	
No	54	1.0	374.3 ± 5.7	3200	
Controls					
Overall	22	3.09	52.2 ± 3.5	811.1	
Male/female	10/12	3.09/15.72	41.6 ± 3.8/63 ± 3.4	201.8/811.1	0.742
Patient/control	66/22	1/3.09	336.6/52.2	3200/811.1	< 0.001

Abbreviations: G. mean = geometric mean, max = maximum, min = minimum, N = number.

### 3.3 Relationship of Total Serum IgE Levels With Associated Factors

#### 3.3.1 Helminth Infection

Of the 66 patients in the study, 43 were screened for helminth infection: 2 out of 43 (4.7%) were positive, 1 for *S. mansoni* and the other for Tania species. Total IgE was > 500 IU/mL for the helminth‐infected patients, which was 514.3 and 3200 IU/mL for S. mansoni and Tania‐positive patients, respectively. The geometric mean total IgE levels of patients with positive and negative helminth infections were 1282.9 ± 3.6 and 294.4 ± 5.6 IU/mL, respectively. There was no significant difference in total serum IgE levels between helminth‐infected and non‐infected patients, although the highest geometric mean total serum IgE level was found in helminth‐infected patients (*p* = 0.237) (Table 4).

**Table 4:** Total IgE levels among helminth‐infected and non‐infected asthmatics.

Factor	*N*	TIgE level	G. mean TIgE level	*p*
Helminth infection				0.237
Positive	2		1282.9 ± 3.6	
*S. mansoni*	1	514.3		
Tania species	1	3200		
Negative	41		294.4 ± 5.6	

Abbreviations: G. Mean = geometric mean, N = number, TIgE = total IgE.

### 3.4 Cholesterol Level

The mean cholesterol level was 188.7 mg/dL for patients, which was significantly higher than that of controls, who had a mean cholesterol level of 166.4 mg/dL (*p* = 0.039). A non‐parametric correlation between total IgE levels and cholesterol levels in patients showed a weak negative correlation with a Spearman's *ρ* of −0.057. The correlation in controls showed a positive, weak correlation with a Spearman's *ρ* of 0.185. The correlations were nonsignificant (*p* = 0.648) and (*p* = 0.411) in patients and controls, respectively.

In this study, total IgE levels > 100 IU/mL were considered high, and < 100 IU/mL were considered low. Only 5 (22.7%) of the controls had serum IgE levels > 100 IU/mL (4 of them had serum IgE levels > 100 IU/mL but < 300 IU/mL, and 1 of them had a serum IgE level > 300 IU/mL, which is 811.1 IU/mL), whereas 50 (75.8%) patients had serum IgE levels > 100 IU/mL. The mean cholesterol level was higher in low IgE groups and lower in high IgE groups of patients, which was negatively associated with IgE levels (odds ratio = 0.995, 95% CI = 0.982–1.009). However, the association was not significant (*p* = 0.507) (Table 5).

**Table 5:** Relationship of cholesterol level and total IgE groups.

Factor	Low IgE group	High IgE group	Total (*M*)	*p*
Cholesterol level^a^ (*M*)				
Patients	194.5	186.9	188.7	0.507
Controls	165	171	166.4	0.808

Abbreviation: M = mean.

a Cholesterol level in mg/dL, low ≤ 100 IU/mL, high ≥ 100 IU/mL.

### 3.5 Asthma Severity

Regarding the severity of asthma, the intermittent group accounted for 8 out of 66 (12.1%), while 35 out of 36 (53%), 11 out of 66 (16.7%), and 12 out of 66 (18.2%) accounted for the mild, moderate, and severe persistent groups, respectively. The geometric mean total IgE level for intermittent, mild, moderate, and severe persistent asthmatics was 399.7 ± 14.9, 305.6 ± 4.2, 567.1 ± 5.9, and 246.8 ± 5.2 IU/mL, respectively.

There was no significant difference in the distribution of total IgE level across severity groups (*p* = 0.324). However, the median levels tended to be higher in intermittent and moderately persistent groups. When patients were categorized into intermittent‐mild and moderate‐severe asthma groups, the moderate‐severe group exhibited geometric mean total IgE level of 367.4 ± 5.6 IU/mL, surpassing the intermittent‐mild group, which had a value of 321.2 ± 5.4 IU/mL (*p* = 0.803). In addition, 36.0% of those with high IgE levels had moderate‐to‐severe asthma, compared to 31.2% of those with low IgE.

### 3.6 Smoking Status

Based on the self‐reported questionnaires regarding their smoking status, four (6.1%) patients were ex‐smokers, who were smoking but stopped, and the others were nonsmokers. The geometric mean of total IgE levels in ex‐smokers (305.4 ± 6.6 IU/mL) was lower compared to nonsmokers (338.7 ± 5.5 IU/mL) (*p* = 0.936).

### 3.7 Relationship of IgE Levels With Other Factors

Patients with and without a family history of asthma had a geometric mean total IgE level of 164.4 ± 3.3 and 374.3 ± 5.7 IU/mL, respectively (*p* = 0.074). In addition, 14.3% of patients with high IgE had a family history compared with 25.0% of patients with low IgE (OR = 0.5; 95% CI = 0.125–1.999). Similarly, patients with hospital admission had a geometric mean total IgE level of 292.1 ± 5.6 IU/mL, while patients without hospital admission had 491 ± 5.0 IU/mL (*p* = 0.216). In addition, 72.0% of patients with high IgE had been previously hospitalized, compared with 75.0% of patients with low IgE (OR = 0.857; 95% CI = 0.236–3.111). The relationship between IgE levels and remaining factors, such as exacerbation, BMI, and physical activity, was not statistically significant.

## 4 Discussion

The result of this study revealed that the geometric mean total IgE level in asthmatics (336.6 ± 5.4 IU/mL) was significantly higher than in controls (52.2 ± 3.5 IU/mL), indicating the central role of total IgE levels in asthma pathogenesis. The finding was in line with previous studies in India [25], Nepal [26], and Iraq [27]. In contrast, a study in Ethiopia showed that the mean level of total IgE was a little higher in cases, but the difference was not statistically significant. The study also demonstrated a uniformly higher concentration of total serum IgE (1345 IU/mL) among patients [28]. Because of the inflammatory process in their airways, asthmatics produce higher amounts of IgE, which accounts for the high levels of total serum IgE seen in the patients [29].

The highest total IgE level found in this investigation was 3200 IU/mL. None of the controls had IgE levels at or above 1000 IU/mL. There was only one control subject with levels ≥ 300 IU/mL, assuming that the upper limit of the IgE level is 300 IU/mL. This recorded level above 300 IU/mL in the controls is due to the fact that the level may sometimes rise in healthy individuals without the disease as well. The outcome thus suggests that a 300 IU/mL cut point may be a useful value for the diagnosis of asthma, particularly the allergic type. This finding was in disagreement with a study in Nepal [26] that reported the highest total IgE level of 3300 IU/mL, found two controls having a total IgE level at or above 1000 IU/mL, and 19 controls having ≥ 300 IU/mL. The possible reasons for the differences might be race, a larger sample size, and the wider age range of controls used in the previous study.

In this study, there was no significant correlation between total IgE level and age, either in the asthma or control groups. However, the lowest median total IgE level was recorded in the 70+ age group of patients. The probable reason for the decreased median total IgE levels observed could be due to a progressive decline of immune function called immunosenescence in aging that results in reduced antibody production [30]. It could also be due to the fact that there was a non‐allergic phenotype of asthma in these groups of patients, though their classification was not confirmed. The result was in agreement with a study done in Germany that showed a significant decrease in total IgE levels with age [31]. On the other hand, a study in Italy concluded that total IgE levels increased with age [30]. The contradictions could be due to variations in sample size, age range, the presence of other underlying allergic diseases, patient selection, and the age of onset of the disease in each study. The variation might also be due to a shift in the inflammatory phenotype towards the Th17 phenotype, in which IgE is often lower [11].

In this study, the geometric mean total IgE level of patients was higher in males as compared to females, whereas in controls, it was higher in females than in males. But the differences were not significant in either group. The possible reason for the variation could be that sex hormones, such as testosterone, could play a role by regulating mast cell activation and Th2 immune responses. Several studies have shown that testosterone has an immunosuppressive effect, which is associated with a decrease in Th2 responses, while estrogens and progesterone are linked to heightened Th2 activity [32, 33, 34]. Similar to this study, a study in Mexico and the United States found no significant difference in total IgE level between genders [35]. In contrast, a study done in Spain [36] and Nepal [26] found significantly higher IgE levels in males than females. The variations could be mainly due to differences in the percentage of male and female study participants.

In the present study, the low rate of positive stool results was not sufficient to detect the association of helminth infection with total IgE levels. However, based on the available data, the study found higher geometric mean total IgE levels in helminth‐infected patients (1282.9 ± 3.6) than in non‐infected patients (294.4 ± 5.6), with a nonsignificant difference. Even though nonsignificant, the higher total serum IgE levels in helminth‐infected patients may be due to the fact that helminth infection induces non‐specific polyclonal IgE and anti‐parasite‐specific IgE production [37]. The nonsignificant association observed could be mainly because of the low prevalence of helminth infection in this study. A number of previous studies showed an increased total IgE level in helminth‐infected asthmatic patients [38, 39]. Furthermore, a study done in Indonesia [40], Bangladesh [41], Latin America [42], and the Philippines [43] showed an association of total IgE levels with helminth infection.

This study disclosed that there was no significant association between serum total IgE level and cholesterol level. Contrary to this result, a study in Japan reported a significant positive association between serum cholesterol levels and total IgE levels [44]. It is well established that cholesterol promotes Th2 immunity and allergic inflammation in rodents [45], supporting the above study. Another mouse model study also states that hypercholesterolemia modulates the Th2 switch of the immune response and markedly increases IgE antibody titers [46]. On the other hand, a study done in Finland reported that participants with elevated IgE had low cholesterol levels [47]. These contradictory results may be explained by factors such as differences in age groups and ethnicities studied, as well as inherent methodological limitations in the study design.

Our analysis of the relationship between total IgE levels and asthma severity found a nonsignificant difference, which is in concurrence with studies in Spain [36] and India [48]. However, studies in the United States and Mexico [35] and in Croatia [49] reported an association of IgE levels with asthma severity. On average, higher IgE levels were associated with moderate to severe asthma in this study, demonstrating how total IgE levels contribute to the pathophysiology of asthma severity. These differences could be mainly due to variations in age groups studied, gender, ethnicity, number of patients in each severity group, and asthma severity classification methods.

The association of smoking with total IgE levels was not ascertained because of the absence of current smokers and the small prevalence of ex‐smokers. Hence, we did not get the necessary statistical power to demonstrate an association. But, with the existing analysis, the geometric mean total IgE levels in ex‐smokers (305.4 ± 6.6) were lower than in nonsmokers (338.7 ± 5.5), with a nonsignificant difference. A study done in Belgium [50] reported that the total IgE level was not increased because of exposure to cigarette smoke. On the contrary, a study done in Korea [51] reported an association between smoking and elevated total IgE levels. In addition, a study done in Korea [52] found enhanced OVA‐specific IgE levels with cigarette smoke.

In this study, a trend toward higher IgE levels was observed in patients without family history, with no hospital admission, with exacerbations, with no physical activity, and with underweight groups. However, none of these variables remained significant. This could possibly be due to recall bias, the length of follow‐up, and the drugs taken by patients.

### 4.1 Limitations

The cross‐sectional nature of the study design might not implicate causal relationships between total IgE levels and associated factors. During recruitment, a higher number of female patients were enrolled as compared to males, and an equal number of patients were not present in each severity group, which could bias the comparison. The age distribution was not fully matched between patients and controls, which could affect the findings of the analysis. The patient's asthma phenotype was not known, which can influence the total IgE level and the analysis.

## 5 Conclusion

The total serum IgE level was significantly higher in asthmatic patients than in apparently healthy controls. It was found that over three‐fourths of patients had high total IgE levels. The assessment of total IgE level could be a direct measurement of airway inflammation in asthmatics, particularly in allergic asthma phenotypes, and should be included in day‐to‐day practice. We did not find a significant difference or association in total IgE levels with helminth infection, cholesterol level, disease severity, smoking status, sociodemographics, or other clinical characteristics. However, total IgE levels tended to be higher in helminth‐infected asthmatics, moderate‐to‐severe asthma groups, and nonsmokers. Higher cholesterol levels were found in the low IgE groups of patients, and future research is required in a larger population to clearly determine the cholesterol association with IgE and its clinical implications. In addition, studies that consider larger sample sizes, specific IgE antibodies, and asthma phenotypes will enrich the findings.

## Author Contributions

Ayenew Assefa conceived the study, ran the lab work, analyzed and interpreted the data, and wrote the paper. Tadelo Wondmagegn and Markos Negash designed, supervised, and analyzed the study and interpreted and reviewed the manuscript thoroughly for its scientific content. All authors have read and approved the final version of the manuscript.

## Conflicts of Interest

The authors declare no conflicts of interest.

## Transparency Statement

The lead author Ayenew Assefa affirms that this manuscript is an honest, accurate, and transparent account of the study being reported; that no important aspects of the study have been omitted; and that any discrepancies from the study as planned (and, if relevant, registered) have been explained.

## Data Availability

The data that support the findings of this study are available from the corresponding author upon reasonable request.
